# Association between Electronic Cigarette Use and Asthma among High School Students in South Korea

**DOI:** 10.1371/journal.pone.0151022

**Published:** 2016-03-04

**Authors:** Jun Ho Cho, Samuel Y. Paik

**Affiliations:** 1 Department of Public Health Administration, Hanyang Women’s University, 17 Haeng-Dong Sungdong-Ku, Seoul 133–793, Republic of Korea; 2 Lawrence Livermore National Laboratory, 7000 East Ave, Livermore, California 94550, United States of America; Research Center Borstel, GERMANY

## Abstract

**Objectives:**

Little is known about health outcomes related to electronic cigarette (EC) use, despite its growing popularity. The aim of this study is to investigate the association between EC use and asthma.

**Methods:**

The study design is a cross-sectional study. A total of 35,904 high school students were included as the final study population. The presence of asthma was based on a student’s self-reported doctor diagnosis of asthma in the past 12 months.

**Results:**

Prevalence rates of asthmatics in ‘current EC users’ (n = 2,513), ‘former EC users’ (n = 2,078), and ‘never EC users’ (n = 31,313), were 3.9% (n = 98), 2.2% (n = 46) and 1.7% (n = 530), respectively. Comparing ‘current EC’ users with ‘never EC’ users, the unadjusted OR for asthma was 2.36 (95% CI: 1.89–2.94). In order to control for the effect of conventional cigarette (CC) smoking, after stratifying the subjects by the three CC smoking categories (never CC, former CC, and current CC), within the ‘never CC’ category, the unadjusted OR for asthma for ‘current EC’ users was 3.41 (95% CI: 1.79–6.49), and the adjusted OR was 2.74 (95% CI: 1.30–5.78). Severe asthma was reflected by the number of days absent from school due to asthma symptoms; current EC users had the highest adjusted OR for severe asthma compared to ‘never EC’ users.

**Conclusions:**

When compared to a reference population of high school students in South Korea, EC users have an increased association with asthma and are more likely to have had days absent from school due to severe asthma symptoms. In conclusion, the results indicate that EC use may be a risk factor for asthma. The results may be useful in developing a scientific basis for the evaluation of a potential health hazard by EC.

## Introduction

Electronic cigarettes (ECs) are battery-powered electronic devices that deliver nicotine aerosols without burning tobacco. The devices consist of a sensor, a battery, and a cartomizer which includes a nicotine cartridge, heating element (atomizer) and glass-fiber wick and reservoir. The glass-fiber reservoirs have a role in holding the nicotine solution [1].

The use of ECs has increased sharply worldwide in recent years. In 2013, EC sales were about $650 million in Europe and about $1.7 billion in the United States [2].In 2013, EC use among adults was reported to be 6.5% in Barcelona, Spain [3] and8.5% in the United States [4]. In 2011, EC use was reported to be 9.4% among Korean adolescents (both current and past users), with 4.5% being current EC users [5]. This was a marked increase from the0.5% (both current and past users) that was reported among Korean adolescents in 2008 [6].

In general, ECs have been used as smoking cessation aids or as alternatives to conventional cigarettes (CCs) [7]. Some studies suggest the EC as contributing to the reduction or cessation of cigarette smoking,[8] while others criticize it as a dangerous product of its own [9, 10]. EC aerosols are known to contain toxicants such as tobacco-specific nitrosamine (TSNA), formaldehyde, and acetaldehyde, though the concentration levels are relatively low [11, 12]. For example, recent studies reported that the levels of toxicants in EC vapors were 9–450 times lower than those in cigarette smoke [13]. However, there are also materials that are unique to ECs, which may pose risk factors specifically for EC users (Table 1).

**Table 1:** Summery for EC components and adverse effects.

EC components	Specific elements detected in EC aerosol or solution [1]	Adverse effects
Inner and outer glass fibers: Silicate particles	Silicon, calcium, aluminum, and magnesium	Inflammatory process in the bronchial tree [14, 15]
Thick copper wire coated with silver, nickel-chromium heating filaments, and others (mouthpiece, solder et. al.)	Copper, silver, nickel, chromium, iron, lead, and manganese	Respiratory distress and diseases [1]
Nicotine cartridge	Nicotine	Asthma [16, 17]
Nanoparticles(<10nm)	Tin, chromium, and nickel	Oxidative stress and inflammation in the lung [18–20]

Asthma is a prevalent chronic disease in Western countries [21] and in the United States [22]. There have been several reports on the adverse effects of CC smoking related to asthma [23]. Similarly, it is reported that smoking is predictive of the development of asthma in allergic adults [24]. However, little is known about EC use with asthma.

To the best of our knowledge, there has never been a large population study assessing the relationship between EC use and asthma, particularly among high school students. It should be noted that the primary objective of this study is to assess the association between EC use and the newly diagnosed asthma (within the past 12 months) in high school students, not the increase in asthma attacks in already-diagnosed asthmatics.

## Methods

### Data

Data was used from The Tenth Korean Youth Risk Behavior Web-based Survey (KYRBWS), a national representative cross-sectional study of 7th to 12th school grade students, from 800 middle and high schools in 2014 [25]. The KYRBWS, which was performed by the Ministry of Education, Ministry of Health and Welfare, and Korean Center for Disease Control and Prevention (KCDC), was reviewed and approved by an institutional review board of KCDC (2014-06EXP-02-P-A), ensuring data quality and compliance with ethical requirements. Only the data from high school students (corresponding to 10^th^ to 12^th^ grades) were included in the final study population since they had much higher rates of EC and/or CC use than data from middle school. The KCDC applied a stratified, two-stage cluster sampling strategy. Four hundred high schools were initially selected from 129 strata identified using a stratified multistage cluster sampling method. Then, 3 classes (1 class per grade) from each school were sampled. Students responded to the online questionnaire anonymously. This Web-based Survey was designed to ensure full participation by allowing students to only proceed to the next page if they fulfilled all questions in the current page. Out of 74,167 students that were given the survey, 72,060 students responded, resulting in an overall response rate of 97.2%. After excluding the middle school students from the survey (approximately half of the respondents), a total of 35,904 students of 37,192 high school students were used as the final study population, resulting in a response rate of 96.5%.

### Outcome definition

Asthma as an outcome was based on student’s self-reported doctor’s diagnoses of asthma. Students were asked the question: ‘In the past 12 months, have you ever been diagnosed with asthma by a doctor?’ (yes/no).Answering yes was classified as ‘asthma.’ In addition, to estimate the association between EC use and severe asthma, an ‘absence from school due to asthma’ variable was assessed by the question ‘In the past 12 months, how many days were you absent from school due to asthma symptoms?’ Response options were then re-classified into four groups: ‘No asthma’, ‘yes asthma, but no absence,’ ‘1–3 day absence,’ and ‘≥4day absence.’

### EC use and CC smoking

EC use was assessed by the question ‘Have you ever used an EC in your life?’ (yes/no). Answering no was classified as ‘never user’. Respondents who answered in the affirmative were asked a follow-up question ‘Have you used ECs in the past 30 days?’(yes/no). Answering yes was classified as ‘current user’ [5] and answering no was classified as ‘former user’. Similarly, cigarette smoking was assessed by the question ‘Have you ever smoked, even one puff in your life?’ (yes/no). Answering no was classified as ‘never smoker’. Respondents who answered in the affirmative were asked a follow-up question ‘In the past 30 days, how many days did you smoke?’ Answering ‘one or more days’ was classified as ‘current smoker’, and answering ‘none’ was classified as ‘former smoker.’

### Socio-demographic and other variables

Gender, high school grade, city size, student’s economic status, residential type, multi-cultural family status, academic performance, overweight status, stress, atopic dermatitis history, allergic rhinitis history, asthma history, attempt to quit smoking, second hand smoke exposure, and cigarette use pattern were investigated in the analyses. Multi-cultural family status was included as a variable due to the recent increase in multi-cultural families in South Korea and its impact on the health of the children. Overweight status was determined by BMI. The BMI is calculated as the body mass divided by the square of the body height, and is expressed in units of kg/m^2^, resulting from weight in kilograms and height in meters. Response options were ‘yes’ if BMI ≥25 kg/m^2^ and ‘no’ if BMI < 25 kg/m^2^. Second hand smoking was assessed by the question ‘In the past week, how many days were you exposed to second hand smoke at home?’ Response options were re-classified into two groups: ‘None’ and ‘more than one day.’

### Statistical analysis

We used sampling information provided in the public use guideline (Korea Centers for Disease Control and Prevention 2015), and performed a statistical analysis of the survey data. First, we used a binary-logistic regression analysis for the association between asthma and all variables, using the forward-stepwise method (included condition: ‘p = 0.05’). As a result of the first step, seven variables were found to be significant and included in the model, including gender, city size, multi-cultural family status, overweight status, second hand smoking at home, atopic dermatitis history, and allergic rhinitis history. In the second step, a variable for CC smoking was added, for a total of eight variables. Next, to find out the changes-in-estimate for each potential confounder of the effect of EC on asthma, we performed multiple logistic regression analyses for each potential confounder. After that, we performed a multiple logistic regression analysis with an interaction term for EC*CC. Next, after stratifying the subjects by the three CC smoking categories (never CC, former CC, and current CC), we analyzed the association between EC use and new asthma diagnosis, using binary-logistic regression models, with adjustment for those selected variables, in models A, B, and C. In addition, to evaluate the effects of EC use on the frequency of students’ absence from school due to asthma, we used multi-nominal logistic regression analyses. In those multi-nominal logistic regression analyses, the ‘no asthma symptom’ was a reference category compared with the ‘1–3 day absence’ and ‘≥4day absence.’ Then, the multi-nominal logistic model controlled for gender, city size, and multi-cultural family status in model B, and the above plus overweight status, second hand smoking at home, atopic dermatitis history, and allergic rhinitis history in model C. All analyses were performed using SPSS 21.0 version software.

## Results

### Population characteristics

A total of 35,904 students of 37,192 high school students were included as the final study population, resulting in a response rate of 96.5% (Table 2).

**Table 2:** Characteristics of the population sample composed of high school students in 2014 (n = 35,904).

Characteristics	No.	(%)
Mean age (y) ± SD		16.4 ±0.9
Gender		
Female	17997	50.1
Male	17902	49.9
High school		
10^th^ grade	11824	32.9
11^th^ grade	12152	33.8
12^th^ grade	11982	33.2
City size		
Large	16232	45.2
Middle	16808	46.8
Small	2864	8.0
Economic status		
High	9898	27.6
Normal	17707	49.3
Low	8299	23.1
Residence type		
With family	33372	92.9
With relatives	364	1.0
With friends in dorm.	2016	5.6
In care facilities.	152	0.4
Multi-cultural family status		
Korean family	34124	95.0
Multi-cultural family	248	0.7
N/A	1532	4.3
School records		
Excellent	3695	10.3
Great	8432	23.5
Good	10558	29.4
Bad	8994	25.1
Worst	4225	11.8
Overweight status		
BMI < 25	30688	85.5
BMI ≥25	4091	11.4
Missing	1125	3.1
Stress		
Never	909	2.5
Rarely	4786	13.3
Moderately	15569	43.4
Much	10664	29.7
Very much	3976	11.1
Atopic dermatitis history		
Not in the last 12 months	33430	93.1
Yes in the last 12 months	2474	6.9
Allergic rhinitis history		
Not in the last 12 months	29406	81.9
Yes in the last 12 months	6498	18.1
Asthma symptom history		
Not in the last 12 months	35229	98.1
Yes in the last 12 months	674	1.9
Attempt to quit smoking in the last 12 months		
No	1367	3.8
Yes	3327	9.3
N/A	31210	86.9
Second hand smoking at home		
None/week	23855	66.4
≥1/week	12049	33.6
Conventional cigarette (CC) smoking		
Never CC smoker	26490	73.8
Former CC smoker	4720	13.1
Current CC smoker	4694	13.1
Electronic cigarette (EC) use		
Never EC user	31313	87.2
Former EC user	2078	5.8
Current EC user	2513	7.0

The mean age of the students was 16.4 (SD: 0.9), and 50.1% (n = 17,997) were female. Most parents were Korean (above 95.0%), and the others were Korean-Chinese, Chinese, North Korean, Vietnamese, Filipino, Japanese, Taiwanese, Mongol, Thai, Cambodian, Uzbekistani, Russian, and others. Overall, 11.4% of the students were overweight and 33.6% were exposed to second hand smoke at home more than one day per week. In addition, 9.3% had attempted to quit smoking in the past 12 months. Approximately, 1.9% (n = 674) students reported that they had been diagnosed with asthma by a doctor in the past 12 months. As for CC smoking, 13.1% (n = 4,694) were current CC smokers, and 13.1% (n = 4,720) were former CC smokers, while for EC use, 7.0% (n = 2,513) were current EC users, and 5.8% (n = 2,078) were former EC users.

### EC use increased odds of asthma diagnosis

We included ‘EC use’ in the models and performed multiple logistic regression analyses for each of the potential confounders, respectively, to find out which factors actually changed the estimates of the effect of EC on asthma (Table 3).

**Table 3:** Results of multiple logistic regression analyses for each potential confounder on the effect of EC on asthma.

Models	Total	No.(%)	Asthma
	No.	(n = 674)	OR (95% CI)
Model 1			
Never EC	31313	530(1.7)	1
Former EC	2078	46(2.2)	1.32(0.97–1.78)
Current EC	2513	98(3.9)	2.36(1.89–2.94)***
Model 2			
Never EC	31313	530(1.7)	1
Former EC	2078	46(2.2)	1.19(0.87–1.61)
Current EC	2513	98(3.9)	2.09(1.67–2.62)***
Model 3			
Never EC	31313	530(1.7)	1
Former EC	2078	46(2.2)	1.31(0.97–1.78)
Current EC	2513	98(3.9)	2.36(1.89–2.93)***
Model 4			
Never EC	31313	530(1.7)	1
Former EC	2078	46(2.2)	1.31(0.96–1.77)
Current EC	2513	98(3.9)	2.26(1.81–2.82)***
Model 5			
Never EC	31313	530(1.7)	1
Former EC	2078	46(2.2)	1.33(0.97–1.82)
Current EC	2513	98(3.9)	2.33(1.85–2.93)***
Model 6			
Never EC	31313	530(1.7)	1
Former EC	2078	46(2.2)	1.27(0.94–1.73)
Current EC	2513	98(3.9)	2.25(1.80–2.80)***
Model 7			
Never EC	31313	530(1.7)	1
Former EC	2078	46(2.2)	1.35(0.99–1.83)
Current EC	2513	98(3.9)	2.35(1.89–2.93)***
Model 8			
Never EC	31313	530(1.7)	1
Former EC	2078	46(2.2)	1.31(0.96–1.78)
Current EC	2513	98(3.9)	2.36(1.89–2.95)***
Model 9			
Never EC	31313	530(1.7)	1
Former EC	2078	46(2.2)	1.07(0.76–1.50)
Current EC	2513	98(3.9)	1.73(1.28–2.34)***

Model 1; unadjusted

Model 2; adjusted for gender only

Model 3; adjusted for city size only

Model 4; adjusted for multi-cultural family status only

Model 5; adjusted for overweight status only

Model 6; adjusted for second hand smoking at home only

Model 7; atopic dermatitis history only

Model 8; adjusted for allergic rhinitis history only

Model 9; adjusted for CC smoking only

***; p < 0.001.

Prevalence rates of asthmatics in ‘current EC users’ (n = 2,513), ‘former EC users’ (n = 2,078), and ‘never EC users’ (n = 31,313), were 3.9% (n = 98), 2.2% (n = 46) and 1.7% (n = 530), respectively. Comparing ‘current EC’ users with ‘never EC’ users (reference population), the unadjusted OR for asthmawas2.36 (95% CI: 1.89–2.94). Comparing ‘current EC’ users with ‘never EC’ users (reference population), the adjusted OR for gender only was 2.09 (95% CI: 1.67–2.62), and the adjusted OR for CC smoking only was 1.73 (95% CI: 1.28–2.34). The CC smoking was the highest factor which affected the effect of EC on asthma, as reflected by the reduction in OR. The gender was the second factor. For all other factors, the changes-in-estimate of the effect of EC on asthma were comparable to that of the unadjusted model.

Using the subset of asthmatics (n = 674), we performed a test to assess the association of EC and an attempt to quit smoking. Among 674 asthmatics, 150 students were CC smokers and 524 students were non-smokers. The result indicated that there was no significant association between EC and an attempt to quit smoking (Table 4).

**Table 4:** Results of logistic regression analyses for assessing the association of EC and an attempt to quit smoking in the subset of asthmatics.

	Total No of CC Smokers(n = 150)	No of CC Smokers who Attempted to Quit Smoking (%) (n = 107)	OR (95% CI) for Attempt to Quit Smoking
Electronic cigarette (EC) use			
Never EC	45	33(73.3)	1
Former EC	26	17(65.4)	0.69(0.24–1.95)
Current EC	79	57(72.2)	0.94(0.41–2.15)

Comparing ‘former EC’ users and ‘current EC’ users with ‘never EC’ users, the ORs for ‘attempt to quit smoking’ were 0.69 (95% CI: 0.24–1.95) and 0.94 (95% CI: 0.41–2.15), respectively.

To determine if there was an interaction effect between EC and CC use on asthma, we performed a logistic regression with an interaction term for EC*CC. Table 5 showed a marginally significant interaction of EC and CC when comparing current versus never smoking (95% CI: 0.20–1.07).

**Table 5:** Results of multiple logistic regression analyses with interaction term for EC*CC among high school students in Korea.

		Asthma (n = 674)		
	Total No.	Model A	Model B	Model C
		OR (95% CI)	OR (95% CI)	OR (95% CI)
Electronic Cigarette (EC)				
Never EC	31313	1	1	1
Former EC	2078	1.10(0.49–2.48)	0.99(0.44–2.24)	0.96(0.42–2.19)
Current EC	2513	3.41(1.79–6.49)***	2.88(1.50–5.51)*	2.77(1.31–5.85)**
Conventional Cigarette (CC)				
Never CC	26490	1	1	1
Former CC	4720	1.11(0.85–1.44)	1.03(0.79–1.34)	0.99(0.75–1.31)
Current CC	4694	1.71(1.25–2.34)***	1.49(1.09–2.05)*	1.47(1.05–2.06)*
EC*CC				
Never EC* Never CC	25962	1	1	1
Former EC* Former CC	710	1.01(0.37–2.74)	1.06(0.39–2.89)	1.16(0.42–3.19)
Former EC* Current CC	1029	0.84(0.33–2.17)	0.94(0.36–2.43)	0.91(0.34–2.40)
Current EC* Former CC	308	0.49(0.19–1.27)	0.53(0.20–1.39)	0.60(0.21–1.71)
Current EC* Current CC	2016	0.43(0.20–0.90)v	0.49(0.23–1.04)	0.46(0.20–1.07)

Model A; unadjusted

Model B; adjusted for gender, city size, multi-cultural family status

Model C; adjusted for the above plus overweight status, second hand smoking at home, atopic dermatitis history, and allergic rhinitis history.

*; p < 0.05,

**; p < 0.01,

***; p < 0.001.

In order to control for the effect of CC smoking, after stratifying the subjects by the three CC smoking categories (never CC, former CC, and current CC), we analyzed the association between EC use and new asthma diagnosis, using binary-logistic regression models, with adjustment for those selected variables, in models A, B, and C (Table 6).

**Table 6:** Results of multiple logistic regression analyses of dependent variables with asthma among high school students in Korea.

	Asthma (n = 674)				
Cigarette use pattern	Total No.	No.(%)	Model A	Model B	Model C
			OR (95% CI)	OR (95% CI)	OR (95% CI)
Never CC					
Never EC	25961	419(1.6)	1	1	1
Former EC	339	6(1.8)	1.10(0.49–2.48)	0.96(0.43–2.17)	0.96(0.42–2.19)
Current EC	189	10(5.3)	3.41(1.79–6.49)**	2.84(1.48–5.44)**	2.74(1.30–5.78)**
Former CC					
Never EC	3702	66(1.8)	1	1	1
Former EC	710	14(2.0)	1.11(0.62–1.98)	1.15(0.64–2.07)	1.20(0.66–2.21)
Current EC	308	9(2.9)	1.66(0.82–3.36)	1.71(0.83–3.50)	1.87(0.89–3.93)
Current CC					
Never EC	1649	45(2.7)	1	1	1
Former EC	1029	26(2.5)	0.92(0.57–1.51)	0.98(0.60–1.61)	0.91(0.54–1.54)
Current EC	2016	79(3.9)	1.45(1.00–2.11)*	1.44(0.97–2.11)	1.30(0.86–1.96)

Model A; unadjusted

Model B; adjusted for gender, city size, multi-cultural family status

Model C; adjusted for the above plus overweight status, second hand smoking at home, atopic dermatitis history, and allergic rhinitis history.

*; p < 0.05,

**; p < 0.01.

The ORs for ‘current EC’ user vs. ‘never EC’ users (reference population) were highest in the ‘never CC’ category, and the second highest ORs were in the ‘former CC’ category, and the lowest ORs were in the ‘current CC’ category. Within the ‘never CC’ category, the unadjusted OR for asthma for ‘current EC’ users was 3.41 (95% CI: 1.79–6.49) in Model A, 2.84 (95% CI: 1.48–5.44) in Model B, and 2.74 (95% CI: 1.30–5.78) in Model C. Within the ‘former CC’ category, the unadjusted OR for asthma for ‘current EC’ users was 1.66 (95% CI: 0.82–3.36) in Model A, 1.71 (95% CI: 0.83–3.50) in Model B, and 1.87 (95% CI: 0.89–3.93) in Model C. Within the ‘current CC’ category, the unadjusted OR for asthma for ‘current EC’ users was 1.45 (95% CI: 1.00–2.11) in Model A, 1.44 (95% CI: 0.97–2.11) in Model B, and 1.30 (95% CI: 0.86–1.96) in Model C.

### EC use increased the odds of school absences due to asthma

In order to control for the effect of CC smoking, after stratifying the subjects by the three CC smoking categories (never CC, former CC, and current CC) to evaluate the effects of EC use on the frequency of students’ absence from school due to asthma, we used multi-nominal logistic regression analyses (Table 7).

**Table 7:** Results of multi-nominal logistic regression analyses on the frequency of students’ absence from school for asthma.

	Absence from school due to asthma symptoms				
Cigarette use pattern	Total No.	No.(%)	Model A	Model B	Model C
			OR (95% CI)	OR (95% CI)	OR (95% CI)
≥4day absence in the past year^a^	35903	89(0.2)			
Never CC					
Never EC	25961	38(0.1)	1	1	1
Former EC	339	1(0.3)	2.03(0.28–14.81)	1.70(0.23–12.53)	1.63(0.22–12.15)
Current EC	189	5(2.6)	18.59(7.23–47.82)***	13.21(4.90–35.64)***	15.42(5.11–46.57)***
Former CC					
Never EC	3702	6(0.2)	1	1	1
Former EC	710	2(0.3)	1.77(0.36–8.77)	1.80(0.35–9.16)	2.09(0.38–11.48)
Current EC	308	2(0.6)	4.03(0.81–20.05)	3.53(0.68–18.42)	1.92(0.20–18.04)
Current CC					
Never EC	1649	10(0.6)	1	1	1
Former EC	1029	5(0.5)	0.80(0.27–2.33)	1.03(0.35–3.09)	0.75(0.23–2.48)
Current EC	2016	20(1.0)	1.65(0.77–3.57)	1.75(0.80–3.83)	1.11(0.45–2.72)
1–3 day absence in the past year^a^	35903	153(0.4)			
Never CC					
Never EC	25961	83(0.3)	1	1	1
Former EC	339	5(1.5)	4.64(1.87–11.52)**	4.04(1.61–10.13)	2.36(0.73–7.63)
Current EC	189	4(2.1)	6.81(2.47–18.79)***	5.67(2.01–16.01)**	5.04(1.52–16.64)**
Former CC					
Never EC	3702	15(0.4)	1	1	1
Former EC	710	2(0.3)	0.71(0.16–3.10)	0.63(0.14–2.76)	0.70(0.16–3.12)
Current EC	308	2(0.6)	1.61(0.37–7.08)	1.35(0.30–6.01)	1.57(0.34–7.16)
Current CC					
Never EC	1649	9(0.5)	1	1	1
Former EC	1029	6(0.6)	1.06(0.38–2.99)	1.11(0.39–3.15)	0.88(0.29–2.65)
Current EC	2016	27(1.3)	2.48(1.16–5.29)*	2.46(1.14–5.33)*	2.23(1.02–4.87)*

Model A; unadjusted

Model B; adjusted for gender, city size, multi-cultural family status

Model C; adjusted for the above plus overweight status, second hand smoking at home, atopic dermatitis history, and allergic rhinitis history.

^a^; reference category for multi-nominal logistic regression analyses on the effects of cigarette use on the frequency of students’ absence from school for atopic asthma was ‘no asthma symptom’ group.

*; p < 0.05,

**; p < 0.01,

***; p < 0.001.

Within the ‘never CC’ group, the OR for ‘more than 4 day absence from school due to asthma symptoms’ was 18.59 (95% CI: 7.23–47.82) in Model A, 13.21 (95% CI: 4.90–35.64) in Model B, and 15.42 (95% CI: 5.11–46.57) in Model C. For the ‘former CC’ group and ‘current CC’ group, the differences were not significant. Within the ‘never CC’ group, the OR for ‘1–3 day absence from school due to asthma symptoms’ was 6.81 (95% CI: 2.47–18.79) in Model A, 5.67 (95% CI: 2.01–16.01) in Model B, and 5.04 (95% CI: 1.52–16.64) in Model C. Within the ‘current CC’ group, the OR for ‘1–3 day absence from school due to asthma symptoms’ was 2.48 (95% CI: 1.16–5.29) in Model A, 2.46 (95% CI: 1.14–5.33) in Model B, and 2.23 (95% CI: 1.02–4.87) in Model C. For the ‘former CC’ group, the differences were not significant. These results suggest that EC use was largely associated with the severity of asthma symptoms, as reflected by the number of days absent from school due to asthma symptoms.

## Discussion

Asthma is known to be associated with inflammation. The results from this study are consistent with recent data reporting the effects of ECs on the lung, with increased airway epithelial inflammation in young people [26].The results are also supported by the recent animal experimental finding that the inhalation of nicotine solution in ECs worsened asthmatic symptoms by increasing inflammatory cells, including eosinophils, into the airways, which resulted in airway inflammation and hyper-responsiveness, possibly driven by the growth in the production of interleukin (IL)-4, IL-5, IL-13 and ovalbumin -specific IgE [27].

The EC cartomizer includes a nicotine cartridge, glass-fiber wick and reservoir, and heating filaments, which are comprised of chromium and nickel. Some or all of these materials may play a role in the pathogenesis of asthma. It is possible that fragments of glass fibers may be inhaled together with nicotine aerosols when inhaling EC aerosols. As a result, the respiratory system may be exposed to glass fiber fragments. A recent study showed that silicon, calcium, aluminum, and magnesium were among the most abundant elements in EC aerosol, and these were found in the silicate beads and fragments of the glass-fiber wick by scanning electron microscopy (SEM) and electron dispersion spectroscopy (EDS) [1]. Dorger et al. [14, 15] reported that the persistence of the glass fibers in the bronchial tree was correlated to the process of macrophagic phagocytosis and production of toxic oxygen radicals and superoxide dismutase (SOD), which contribute to the inflammatory process. The metals that are present in EC aerosols may also promote the pathogenesis of asthma. The concentrations of nine of eleven elements in EC aerosol were higher than or equal to the corresponding concentrations in CC [1]. The concentrations of four elements (sodium, iron, aluminum, nickel) were higher in EC aerosol, and the concentrations of five elements (copper, magnesium, lead, chromium, and manganese) were roughly equal to their levels in CC smoke. All of the metals in EC aerosols can adversely affect the respiratory system [1]. For example, inhalation of sodium may cause lung irritation, shortness of breath, and bronchitis. Iron may cause respiratory irritation, metal fume fever, siderosis, and fibrosis. Aluminum may cause asthma and pulmonary fibrosis. And nickel may cause chronic bronchitis, reduced lung function, lung inflammation, lung/nasal sinus cancer, and pulmonary fibrosis. Nicotine, a primary constituent of ECs, may also contribute to the pathogenesis of asthma. Among children diagnosed with asthma, concentrations of nicotine in the settled dusts from the bedrooms were significantly higher than in the controls [16]. Also, soluble components of EC, including nicotine caused dose-dependent loss of lung endothelial barrier function, were associated with oxidative stress and brisk inflammation [17]. Finally, a recent study has shown that particle analysis of EC aerosol revealed the presence of nanoparticles (< 100 nm in diameter) composed of tin, chromium, and nickel [1]. Numerous studies have shown nanoparticles to cause oxidative stress and inflammation in the lung and cardiac tissues of animals and therefore may play a role in causing asthma [18–20]. The asthma-genic potential of nanoparticles is considered in qualitative risk assessment tools be one of many factors that can contribute to nanoparticle toxicity [18]. Therefore, these materials, many of which are unique in ECs, may be why EC users indicated higher risk rates than never EC users for the number of days absent from school due to asthma symptoms.

This study has a number of limitations. Due to it being a case-crossover study, the study cannot establish a cause-and-effect relationship between EC use as a biological factor and the onset of asthma. It is also acknowledged that recall bias is a relevant source of bias in case-crossover studies due to the reliance on self-reported doctor diagnoses of asthma rather than actual hospital records. It is also recognized that responding affirmatively to the question of EC use in the past 30 days may not necessarily reflect current EC use. Another limitation of the study is that the numbers of current EC users is relatively small. The reliability of statistical tests would be improved when the numbers of current EC users increase. The use of school day absences as a measure of asthma severity is also somewhat simplistic. In the future, medical records such as dyspnea, medication use, or hospital admittance need to be considered to more accurately represent the severity of asthma. Finally, because EC is frequently used for smoking cessation, students already diagnosed with asthma may have started using ECs in an attempt to quit smoking to prevent further exacerbation of asthma, resulting in reverse causation. However, there was no significant association between EC and an attempt to quit smoking in the subset of asthmatics in this study. Despite these limitations, this was a large representative and anonymous population study, which was designed systematically, and showed a response rate of over 96%.

## Conclusions

Based on the survey data, even after adjusting for potential confounders, EC use showed increased odds of being diagnosed with asthma when compared to the reference population. EC use also showed increased odds of asthma severity, represented by days absent from school due to asthma symptoms. Also, this study discussed the possible causes for the higher association with asthma in EC users, including the presence of glass-fibers, metals, nicotine and nanoparticles in EC aerosols. In conclusion, the results indicate that EC use may be a risk factor for asthma. The results may be useful in developing a scientific basis for the evaluation of a potential health hazard by EC.

## Abbreviations

EC: electronic cigarette
CC: conventional cigarette
